# Proteomic Analysis of Hepatic Tissue of *Cyprinus carpio* L. Exposed to Cyanobacterial Blooms in Lake Taihu, China

**DOI:** 10.1371/journal.pone.0088211

**Published:** 2014-02-18

**Authors:** Jinlin Jiang, Xiaorong Wang, Zhengjun Shan, Liuyan Yang, Junying Zhou, Yuanqin Bu

**Affiliations:** 1 Nanjing Institute of Environmental Sciences/Key Laboratory of Pesticide Environmental Assessment and Pollution Control, Ministry of Environmental Protection, Nanjing, People's Republic of China; 2 State Key Laboratory of Pollution Control and Resource Reuse, School of the Environment, Nanjing University, Nanjing, People's Republic of China; National Center for Biotechnology Information (NCBI), United States of America

## Abstract

With the rapid development of industry and agriculture and associated pollution, the cyanobacterial blooms in Lake Taihu have become a major threat to aquatic wildlife and human health. In this study, the ecotoxicological effects of cyanobacterial blooms on cage-cultured carp (*Cyprinus carpio* L.) in Meiliang Bay of Lake Taihu were investigated. Microcystins (MCs), major cyanobacterial toxins, have been detected in carp cultured at different experimental sites of Meiliang Bay. We observed that the accumulation of MCs in carp was closely associated with several environmental factors, including temperature, pH value, and density of cyanobacterial blooms. The proteomic profile of carp liver exposed to cyanobacterial blooms was analyzed using two-dimensional difference in-gel electrophoresis (2D-DIGE) and mass spectrometry. The toxic effects of cyanobacterial blooms on carp liver were similar to changes caused by MCs. MCs were transported into liver cells and induced the excessive production of reactive oxygen species (ROS). MCs and ROS inhibited protein phosphatase and aldehyde dehydrogenase (ALDH), directly or indirectly resulting in oxidative stress and disruption of the cytoskeleton. These effects further interfered with metabolic pathways in the liver through the regulation of series of related proteins. The results of this study indicated that cyanobacterial blooms pose a major threat to aquatic wildlife in Meiliang Bay in Lake Taihu. These results provided evidence of the molecular mechanisms underlying liver damage in carp exposed to cyanobacterial blooms.

## Introduction

The famous scenic destination of Lake Taihu, located on the border of Jiangsu and Zhejiang provinces in Eastern China, is the largest lake south of the Yangtze Delta plain (N30°56′–31°34′, E119°54′–120°36′) and the third largest freshwater lake in China. Despite efforts to reduce the contamination of the lake, pollution has continued for decades, associated with the development of industry and agriculture in this area. A large amount of agricultural, mining, and industrial wastewater and living sewage has been directly discharged into the lake, dramatically increasing nitrogen, phosphorus, heavy metals and other pollutants, leading to frequent outbreaks of cyanobacterial blooms.

Meiliang Bay, located on the northwest side of Lake Taihu, is 120 km^2^ in area and highly polluted due to eutrophication. The bay is shallow with a low annual average water change (<2), making this area a trap for pollutants. Eutrophication in Meiliang Bay is extremely serious, and outbreaks of cyanobacterial blooms have occurred from May to October since 1990, particularly in July and August. Microcystins (MCs) are cyanobacterial toxins that are a serious threat to drinking water and recreational lakes worldwide. In 2005, 6.66 µg L^−1^ and 2.71 µg L^−1^ of microcystins were detected, respectively, in samples obtained from Meiliang Bay and Wuli Lake [1]. In May of 2007, cyanobacterial bloom outbreaks resulted in the shutdown of the water system in Wuxi City.

After an outbreak of cyanobacterial blooms, the death of algal cells releases a large number of toxins. Microcystins (MCs) are one of most widely distributed and dangerous cyanobacterial toxins. MCs poison aquatic organisms and are a direct threat to human health [2]. Therefore, it is important to examine the toxicological effects of microcystins and develop strategies to eliminate the causative pollutants. In recent years, many studies have focused on molecular biomarkers as indicators for the early diagnosis of pollution exposure and ecological risk [3]–[5]. Extensive efforts have been made to study reactive oxygen species (ROS) and antioxidant system indicators [6]. The generation of ROS induced by cyanobacterial toxins is considered one of the most important mechanisms resulting in the poisoning of organisms. However, it is difficult to detect these radicals, such as ·OH, O_2_^•−^ and H_2_O_2_, due to their low concentration, short life span and extremely high activity *in vivo*. In recent years, electron paramagnetic resonance (EPR) technology has become the most direct and effective tool for the detection of free radicals in studies concerning cyanobacterial toxins [5]. Notably, the results obtained from studies of reactive oxygen species or other single indicators do not explain the toxicological mechanisms of certain pollutants, therefore other influences, such as individual sensitivity, the types of pollutants, and changes in the environment, should be considered [7]. Moreover, the use of ecotoxicological methods is needed to screen key indicators in various biological pathways of organisms, which might be affected by cyanobacterial blooms.

In recent years, genomics, transcriptomics, proteomics, and metabolomics have been widely used in the study of ecotoxicology [8]. Proteomics involves the large-scale study of proteins and their biological functions in a whole organism, and the obtained results of these studies provide a platform to determine the mechanisms of toxicity at the molecular level. Recent studies concerning pollutants from cyanobacterial blooms have focused on traditional toxicology and genomics assessments and biochemical index analyses, with little regard to proteomics. Moreover, it is unknown whether cyanobacterial blooms induce changes in protein expression in the fish liver, reflecting the complex effects of cyanobacterial blooms on fish, as this organ accumulates the most toxic MCs.

In the present study, we investigated the effects of cyanobacterial blooms on the ecotoxicology of the common cage-cultured carp in Meiliang Bay compared with fish living in Xukou Bay, which has a higher water quality, and fish living in the laboratory. The expression of hepatic proteins in the fish from the two bays and laboratory was studied using 2D-DIGE combined with mass spectrometry to elucidate the molecular mechanisms underlying the fish liver damage caused by cyanobacterial blooms.

## Materials and Methods

### Ethics Statement

This study was approved by the Animal Ethics Committee of the Nanjing Institute of Environmental Sciences, Ministry of Environmental Protection. The institute does not issue a number or ID to any animal study, but the ethical committee approved and helped guide the animal use in this study. The field study in Lake Taihu was approved by the General Office of Lake Taihu Water Pollution Prevention and Control. No endangered or protected fish species was sampled in this study.

### *In situ* experimental sites

Lake Taihu is the third largest freshwater lake in China, with an area of 2338 km^2^ and an average depth of 1.9 m. Additional information about Lake Taihu has been detailed elsewhere [9]. During the past decades, outbreaks of cyanobacterial blooms have frequently occurred in this area due to the increased population in areas near the lake, leading to intense agricultural and industrial wastes [10].

**Figure 1:**
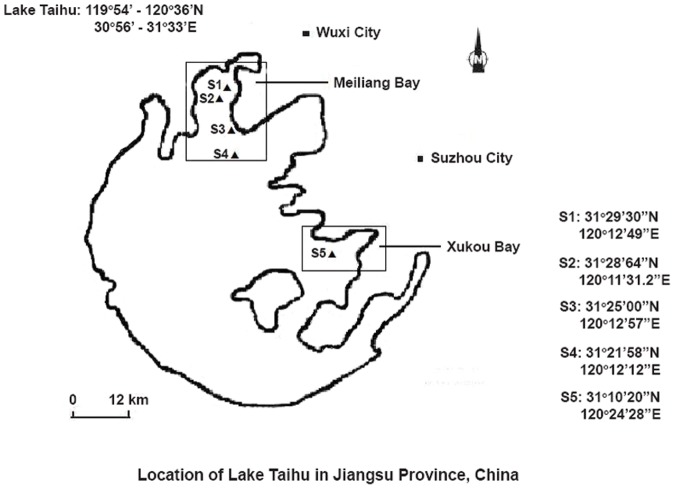
*In-situ* experiment sites.

Experimental sites in Meiliang Bay were S1 (31°29′30″N 120°12′49″E), S2 (31°28′64″N 120°11′31.2″E), S3 (31°25′00″N 120°12′57″E), and S4 (31°21′58″N 120°12′12″E), encompassing an area of 125 km^2^. Serious outbreaks of cyanobacterial blooms (microcystins are the dominant species of these blooms) occur frequently in these areas [11]. Experimental site S5 (31°10′20″N 120°24′28″E), located in Xukou Bay, was chosen as a control site because this region is macrophyte dominated and eutrophic levels are low. The *in situ* experiment sites are shown in Figure 1.

### Experimental design

One hundred and fifty carp (approximately 6 months of age), with an average body length of 13.81±1.09 cm and weight of 30.56±3.99 g, were obtained from the research station of Freshwater Fisheries Research Center, Chinese Academy of Fishery Sciences. These fish were acclimated to laboratory conditions for 5 days with 100 L of dechlorinated tap water. The total mortality of these fish was less than 5%. After acclimation, the carp were randomly divided into five groups (twenty carp per group) and released in a closed net cage and cultured in Lake Taihu for 14 days at different experiment sites (S1–S5). The net cage, with a cylindrical shape of Φ80 cm×110 cm and a mesh size of 4 mm×4 mm, was hung vertically in the water. The net cage was placed approximately 0.5 m from the top and bottom of the lake. The *in situ* study was performed from July 11–24, 2009. The control carp were maintained in the Lake Taihu laboratory (Chinese Academy of Sciences) in 100 L of dechlorinated tap water (10 individuals per aquarium) for 14 d. During acclimation and laboratory testing, a 12-h/12-h day/night photoperiod was maintained, and the water was constantly aerated. Every 48 h, one half of the water was renewed. The fish were fed daily before or during laboratory testing. The water quality parameters during testing are shown in Table 1.

**Table 1:** The physic-chemical parameters of the water from different experiment sites.

Experimental sites	S1	S2	S3	S4	S5	Lake Taihu laboratory
GPS location	31°29′30″N, 120°12′49″E	31°28′64″N, 120°11′31.2″E	31°25′00″N, 120°12′57″E	31°21′58″N, 120°12′12″E	31°10′20″N, 120°24′28E	–
Temperature, °C	33.21	33.10	33.89	32.70	31.11	23.92
Depth, m	2.38	2.50	1.75	2.55	1.71	n.a.^a^
pH	9.47	9.12	8.76	8.93	8.49	8.00
DO, mg/L	15.03	12.32	8.62	11.06	7.90	8.29
Electronic conductivity, ms/cm	0.508	0.544	0.564	0.509	0.580	0.728
Turbidity, NTU	44.7	42.1	73.5	50.8	31.9	6.95
Chl a, ug/L	10.2	9.0	13.1	22.7	4.3	1.9
TN/NH_4_^+^-N/NO_3_^−^-N/NO_2_^−^-N, mg/L	0.95/0.23/0.232/0.016	1.31/0.266/0.207/0.017	3.78/0.432/0.302/0.007	0.37/0.200/0.926/0.004	1.72/0.135/0.771/n.d.^b^	n.a.
TP/DTP/DP, µg/L	192/159/12.00	165/129/21.73	233/60/7.70	95/30/2.49	75/32/4.52	38.9/n.d./20.8
Cyanobacteria cell, ×10^5^ cell/mL	2.13±0.85	1.47±0.53	2.35±0.22	1.05±0.36	0.34±0.12	n.d.
MC-LR/MC-RR concentration, µg/L	0.402/0.307	0.534/0.490	0.362/0.268	0.334/0.184	0.016/n.d.	n.d./n.d.
Heavy metal (Cu/Cd/Cr/Pb/Zn), µg/L	10.6/0.020/2.36/0.76/6.66	6.35/0.016/2.19/0.92/6.61	9.96/0.018/2.21/0.51/8.02	6.29/0.025/2.21/0.69/6.27	6.93/0.022/1.83/0.110/23.5	n.a.

a n.a. = not analyzed.

b n.d. = un-detectable.

The water was sampled every three days from July 11–24, 2009. All data are presented as the means of five water samples.

The fish collected were killed by a sharp blow on the head and then dissected on ice immediately. All efforts were made to minimize suffering. The livers were quickly removed and immediately frozen in liquid nitrogen before storage at −80°C until further analysis. The other tissues (muscle, gill and intestine) were stored at −20°C, and subsequently used to determine toxin content.

### Water samples collection and physicochemical analysis

The samples were collected at two depths (0.5 m from the top and bottom of the lake) every three days from July 11–24, 2009. The water temperature, pH, dissolved oxygen (DO), electronic conductivity and total dissolved solids (TDS) were detected *in situ* using YSI 6600. Chlorophyll-a (Chl-a) was measured according to methods of APHA et al. [12]. The cyanobacteria were stained with Lugol's solution and methylene blue and subsequently counted under a ZEISS fluorescence microscope. The nutrients were measured, including total nitrogen (TN), ammonium nitrogen (NH^4+^-N), nitrate nitrogen (NO_3_^−^-N), nitrite nitrogen (NO_2_^−^-N), total phosphorus (TP), total dissolved phosphorus (DTP) and dissolved inorganic phosphorus (DP), according to the *Standard Methods for the Examination of Water and Wastewater* (fourth edition) [13]. The heavy metal (Cu, Cd, Cr, Pb and Zn) content of the water samples was measured using Sciex Elan 9000 ICP/MS (Perkin-Elmer, USA).

For extraction and determination of the MCs in the water samples, 0.5 to 1.0 L of each sample was filtrated using glass microfiber filters (47 mm, 1.2 µm, Whatman, England), and concentrated through a ODS-C18 solid phase extraction cartridge (Agilent AccuBOND, USA), followed by washing with 20% methanol. The MC-LR or MC-RR in the cartridge was eluted with 90% methanol containing 0.1% trifluoracetic acid (TFA). The eluent was dried through rotary evaporation and reconstituted with methanol. The determination of MCs was performed according to the methods as described by Aranda-Rodriguez et al. [14] and Jiang et al. [15] using HPLC (Agilent 1100 Series, Agilent Technologies, USA) on a Zorbax Eclipse SB-C18 column (250 mm×4.6 mm, 5 µm, Agilent Technologies).

The environmental parameters of the experiment site are shown in Table 1. All data were presented as the means of five water samples. Person's linear correlation was used to analyze the relationships between environmental parameters.

### Detection of MCs in various fish tissues

To extract the toxins from the tissue, the fish were sacrificed and the muscle, gill, liver, and intestine were excised, weighed, and immediately frozen. The MC extraction was performed according to the description of Jiang et al. [16]. The extracts were quantitatively analyzed using an ELISA (Microcystin plate kit, Institute of Hydrobiology, Chinese Academy of Sciences) according to the manufacturer's instructions. The sensitivity of the ELISA was 0.1 µg L^−1^. The MC concentration was expressed as per gram of dry mass (DW).

### ROS trapping and EPR measurement

ROS levels were determined using α-phenyl-N-tert-butylnitrone (PBN) as spin trap reagent, followed by electron paramagnetic resonance (EPR) analysis as described by Luo et al. [17]. The liver was homogenized in ice-cold 50 mM PBN in DMSO using a glass homogenizer. The homogenates were transferred to a quartz capillary tube and immediately stored in liquid nitrogen for subsequent EPR analysis. All operations were performed in a sealed box filled with nitrogen. The EPR spectra were recorded using a Bruker EMX 10/12 X-band spectrometer (Bruker, Germany) as previously described by Luo et al. [17]. The height of the second peak of the EPR signal was interpreted as the intensity of •OH in liver tissues. All samples were performed in quadruplicate. The data were assessed for normality and transformed when necessary to meet the assumption of the normal distribution. The data were compared using one-way ANOVA. Duncan test was used to determine the significant difference between groups. Statistical analyses were performed with SPSS 16 (SPSS Inc., USA). The data were expressed as means ± standard deviation (SD).

### Protein preparation and CyDye labeling

Total protein was extracted from fish liver using lysis buffer (pH 8.5), containing 7 M urea, 2 M Thiourea, 30 mM Tris, 4% (w/v) CHAPS, 10 mM PMSF and 1% protease inhibitor cocktail (Sigma-Aldrich, St. Louis, MO). The protein concentration was determined using a 2-D Quant protein assay kit (GE Healthcare, Uppsala, Sweden). The liver samples used for DIGE analysis were obtained from fish cultured in the laboratory (control group), Xukou Bay S5 (T1 group), and Meiliang Bay S1 (T2 group). The experiment sites S1 and S5 were chosen based on distinct differences in water eutrophication (Table 1). The extracted protein from three randomly selected liver samples were pooled, yielding three protein samples. The samples were subsequently purified using a 2-D Clean-up kit (GE Healthcare). Equal amounts of protein from the different groups were labeled with CyDye Fluor minimal dyes (GE Healthcare) according to the manufacturer's instructions. To create an internal standard (IS), aliquots of the sample in each dataset containing equal amounts of protein were pooled and labeled with Cy2. A total of 50 µg of protein from the T1, T2, and control groups were labeled with 400 pmol of Cy2, Cy3, or Cy5, respectively. The labeled mixtures were combined according to Table S1 and were then adjusted to 450 µL with isoelectric focusing (IEF) rehydration buffer (pH 4–7) containing 7 M urea, 2 M Thiourea, 2% (w/v) CHAPS, 20 mM DTT, and 0.5% IPG buffer, with a trace amount of bromophenol blue.

### 2-D DIGE and image analysis

The labeled samples were loaded onto Immobiline Dry Strips (24 cm, linear pH gradient from pH 4–7, GE Healthcare). The IPG strips were rehydrated at 40 V for 5 h, followed by 100 V for 6 h, and IEF was subsequently conducted for a total of 69.8 kVhr on a Multiphor II System (GE Healthcare). After completion of the IEF analysis, the strips were equilibrated and applied to 12.5% polyacrylamide gels. The SDS-PAGE was performed using Ettan™ Daltsix equipment (GE Healthcare) at 15°C. All electrophoresis procedures were performed in the dark and ran in duplicate. The gels were scanned using a Typhoon™ Variable Mode Imager (GE Healthcare) at a resolution of 100 µm, followed by silver staining. The gel images were analyzed using DeCyder 6.5 software (GE Healthcare). The biological variation analysis mode (BVA) was used to detect the significant between-group differences across all gels. Data was statistically analyzed using Student's *t*-test, and *p*<0.05 was considered significant.

### In-gel trypsin digestion and protein identification through MALDI-TOF

The protein spots from the silver-stained gels were excised and transferred to V-bottom 96-well plates and destained with 100 µL of 15 mM potassium ferricyanide and 50 mM sodium thiosulfate (1∶1) for 20 min, followed by digestion overnight with 12.5 ng/µL trypsin in 20 mM ammonium bicarbonate at 37°C. The samples were dissolved in 0.1% TFA in 50% CAN, applied onto the target and subsequently dried under nitrogen. The peptides were eluted onto the target using 0.7 µL of matrix solution (α-cyano-4-hydroxy-cinnamic acid in 0.1% TFA in 50% CAN). The samples were air-dried and analyzed using a 4700 MALDI-TOF Proteomics Analyzer (Applied Biosystems, Framingham, MA, USA). MALDI-TOF mass spectra were recorded with a Micromass Tofspec E MALDI time of flight mass spectrometer in reflectron mode. The data from the PMF and MALDI-TOF MS/MS were analyzed using MASCOT software (Matrix Science, London, UK). Based on combined MS and MS/MS spectra, mascot scores greater than 64 were considered statistically significant (*p*<0.05). The individual MS/MS spectra with a statistically significant (confidence interval >95%) best ion score (based on MS/MS spectra) were accepted. The identified proteins were matched to the Gene Ontology (http://www.geneontology.org/) and KEGG (http://www.genome.jp/kegg/) databases.

### Quantitative PCR and Western blot

Three proteins [valosin containing protein (VCP), mitochondrial ATP synthase beta subunit (MASb) and glutathione peroxidase (GP)] were chosen for further analysis using quantitative real-time PCR. 18S rRNA (18 S) was chosen as an internal control. Differences in the mRNA expression levels were calculated using the 2^−ΔΔCt^ method [18]–[20]. The Western blots were probed with two antibodies against beta-tubulin and heat shock protein70 (HSP70). The detailed methods for the quantitative PCR and Western blot analysis are given in the supplemental text S1. Statistical significance was determined using a one-way ANOVA, followed by the Duncan multiple range test (SPSS, Inc., Chicago, IL). The data are presented as the means ± SD and *p*<0.05 was considered significant.

## Results

### *In situ* water environment

Meiliang Bay, located northwest of Lake Taihu, has advanced eutrophication due to the low water exchange capacity and shallow depth. A large accumulation of cyanobacterial blooms were observed at experiment sites S1, S2, S3, and S4 in Meiliang Bay, while no cyanobacterial blooms were observed in the S5 location in Xukou Bay, which is dominated by submerged lush plants. The correlation analysis indicated that the density of microcystis was highly correlated with the level of TP and MC-RR (*p*<0.01), the water temperature and the level of MC-LR (*p*<0.05).

### Accumulation of MCs in different tissues of the fish

**Figure 2:**
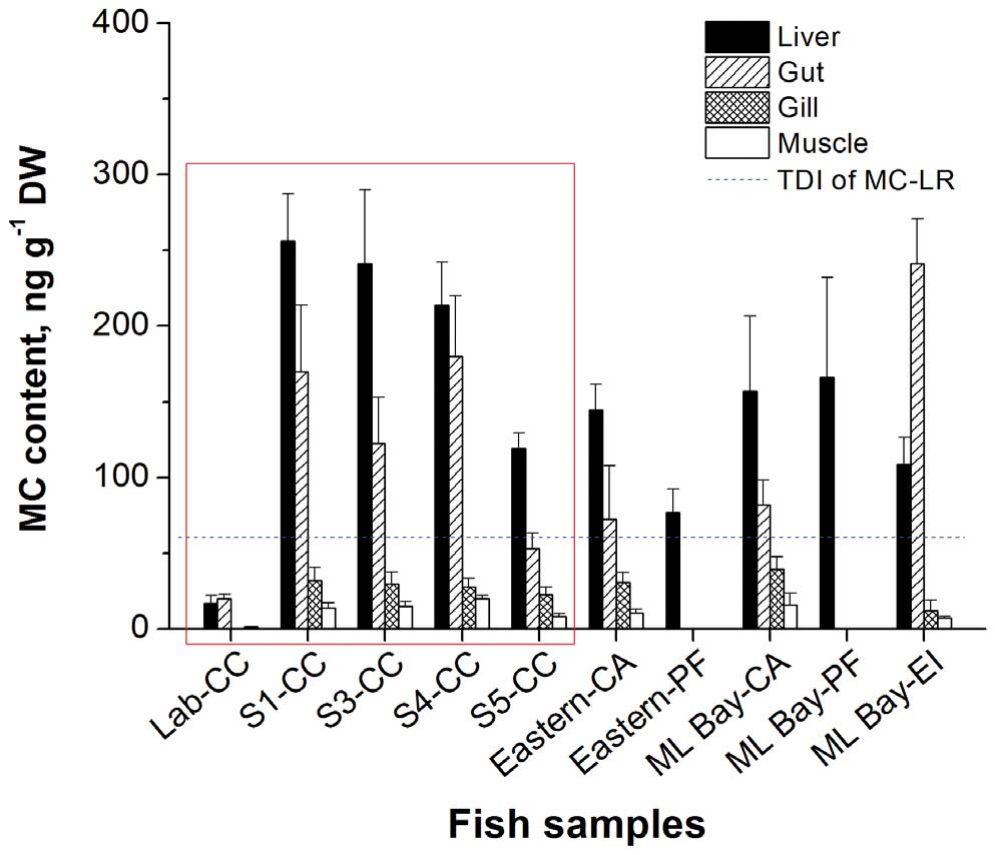
MC content (ng g^−1^ DW) in the organs/tissues (muscle, gill, intestine, liver) of fish. The MC content was determined using ELISA (n = 4). Lab-CC represents carp (*Cyprinus carpio*) cultured in laboratory for the control group. S1-CC, S3-CC, S4-CC, and S5-CC represent the experimental groups for carp cultured in net cages located at experiment sites S1, S3, S4 and S5, respectively. Eastern-CA represents carp (*Carassius auratus*) captured in the eastern part of Lake Taihu (weight of 49.16±10.89 g; length of 15.69±1.28 cm), Eastern-PF represents *Pelteobagrus fulvidraco* captured in the eastern part of Lake Taihu (weight of 24.44±5.90 g; length of 13.80±1.15 cm), ML Bay-CA represents carp captured in Meiliang Bay (weight of 33.4±4.24 g; length of 12.75±0.35 cm), ML Bay-PF represents *Pelteobagrus fulvidraco* captured in Meiliang Bay (weight of 69.50±40.10 g; length of 18.9±3.54 cm), and ML Bay-EI represents *Erythroculter ilishaeformis* captured in Meiliang Bay (weight of 19.95±2.90 g; length of 15.50±0.71 cm).

As shown in Figure 2, MCs were detected in carp from different experiment sites. The carp from the S2 site were lost due to storms. A trace amount of MCs were also detected in the control group, potentially reflecting pollution from farm runoff (MC was not detected in the gill). MC accumulation in the different tissues of cage-cultured carp were observed in the liver>intestine>gill>muscle, indicated with red boxes. The accumulation of MCs was significantly higher in carp from the S1, S3 and S4 sites than from the S5 site and the control group, with the highest accumulation of MCs observed in carp from S1. The correlation analysis indicated that MC accumulation in the liver was significantly correlated with water temperature, pH, electronic conductivity, turbidity, algal density, and the levels of MC-LR, MC-RR and chromium (*p*<0.05). MC accumulation in the intestine was also significantly correlated with water temperature, pH, electronic conductivity, and the levels of MC-LR and lead (*p*<0.05). MC accumulation in the gill was significantly associated with water temperature, electronic conductivity, and chromium concentration (*p*<0.05). MC accumulation in the muscle was significantly positively correlated with water temperature, conductivity, and Chl *a* concentration (*p*<0.05). Notably, the high MC accumulation in carps cultured in Meiliang Bay, which had a relatively high algal density, might be associated with high algal intake, as numerous algae were observed in the intestines of these fish.

The MC level was higher in wild fish from Meilang Bay compared with the eastern part of Lake Taihu. The variation of the MC level in the intestine might reflect the presence of un-digested food. Notably, MCs were also detected in the carnivorous fish species *Pelteobagrus fulvidraco* and *Erythroculter ilishaeformis*, suggesting that MCs can move through the food chain.

### Effects of cyanobacterial blooms on ROS levels in the carp liver

**Figure 3:**
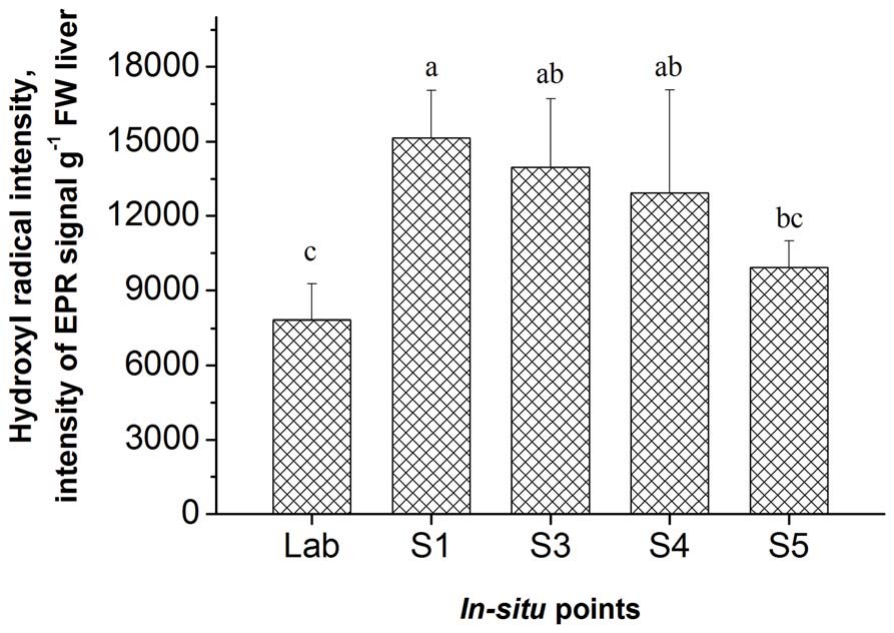
EPR signal intensity of PBN-•OH in the liver of *C. carpio* from lab cultures and *in-situ* experiment sites. The data are shown as the means ± SD (n = 4). The same letter indicates no significant difference between the groups. Different letters indicate significant differences between the groups with *p*<0.05.

Carp were cultured for 14 days in different experiment sites. As shown in Figure 3, the •OH levels in liver were significantly higher in carp cultured at sites S1, S3 and S4 than in carp cultured at the S5 site and in the control group (*p*<0.05). The •OH levels were significantly correlated with the MC accumulation in the fish intestine and gills (*p*<0.05) and particularly in the fish liver (*p*<0.01). Moreover the •OH levels were also associated with water temperature, pH, algal density, and the levels of MC-LR/RR and chromium (*p*<0.05, *p*<0.01).

### Protein expression analysis using 2D-DIGE

**Figure 4:**
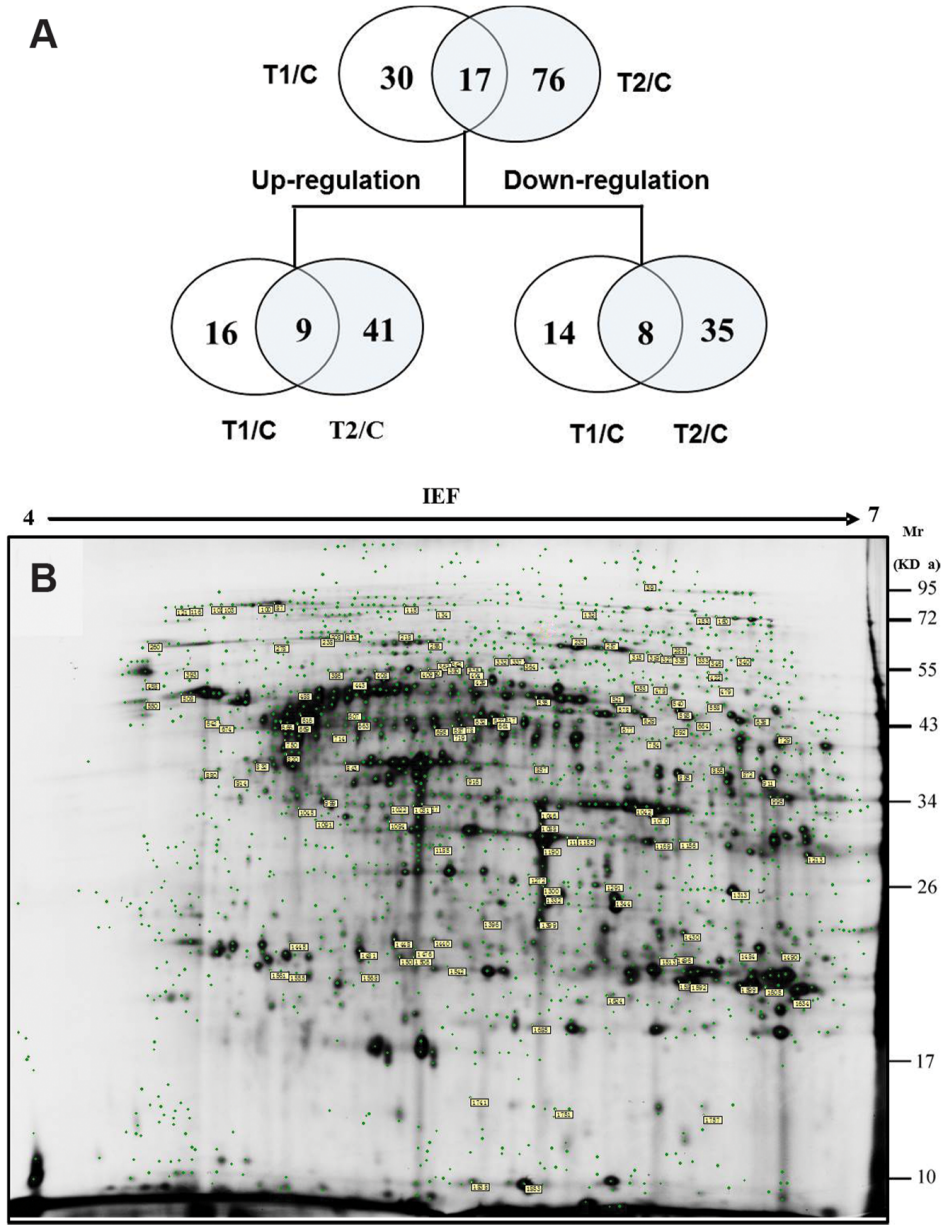
Representative 2D-DIGE gel of the differential expression of hepatic proteins in *C. carpio*. (A) Differential expression patterns of hepatic proteins detected in T1, T2 and control groups analyzed using Decyder software. (B) 2-D DIGE gray-scale image of liver protein expression (Cy2-labeled internal standard). Differentially expressed proteins are indicated with boxes containing the master number.

Protein was efficiently separated using 2D-DIGE (Figure S1). The carp were cultured in the laboratory (control (C)), Xukou Bay (T1), or Meiliang Bay (T2). A total of 148 spots were detected with significant difference using DeCyder™ image analysis software (Figure 4A). A comparison of the T1 and C groups revealed 47 different spots, with 25 spots showing up-regulation. Among the 93 spots different spots detected between T2 and C, 50 spots showed up-regulation. A comparison of T1 to T2 revealed 43 spots, with 27 spots showing up-regulation. A total of 106 spots were analyzed using MALDI TOF mass spectrometry, and 57 proteins were successfully identified (Table 2). The distribution of identified proteins was shown in Figure 4B.

**Table 2:** Classification of the differentially expressed proteins identified in the liver of field treatment fish compared with the fish in laboratory.

Master No. on gel^a^	Protein name	Accession No.^b^	Pep. Count^c^	Protein score^d^	Theoretical Mr (kDa)/pI	Fold change^e^			Functional category^f^
						T1/C	T2/C	T2/T1	
**Metabolism (25)**									
***Amino acid metabolism (10)***									
885	Fumarylacetoacetase	GI:41054569	5	132	38729.4/6.21	**−3.61** *	−1.22	2.96	aromatic amino acid family metabolic process/metabolic process
754	Homogentisate 1,2-dioxygenase	GI:10441585	5	105	44396.3/6.37	−4.64	**−2.41** *	1.93	L-phenylalanine catabolic process/oxidation reduction/tyrosine metabolic process
1430	Histidine ammonia-lyase	GI:148234062	5	106	72081.1/6.15	1.03	**2.09** *	**2.03** *	KEGG pathway: histidine metabolism/metabolic pathways/nitrogen metabolism
677	Hypothetical protein LOC556744	GI:205830395	3	67	74579/6.19	1.29	**2.79** *	2.17	histidine catabolic process
1046	Methionine adenosyltransferase I, alpha	GI:41054081	10	250	43261.9/6.32	1.11	**2.13** *	1.92	one-carbon metabolic process
627	Phenylalanine hydroxylase	GI:41054599	7	122	51321.8/5.6	−1.17	1.24	**1.46** *	L-phenylalanine catabolic process/aromatic amino acid family metabolic process/metabolic process/oxidation reduction/response to chemical stimulus
661	Phenylalanine hydroxylase	GI:41054599	8	142	51321.8/5.6	−1.25	**−1.56** *	−1.24	L-phenylalanine catabolic process/aromatic amino acid family metabolic process/metabolic process/oxidation reduction/response to chemical stimulus
632	Phenylalanine hydroxylase	GI:41054599	7	189	51321.8/5.6	1.06	**−1.82** *	−1.92	L-phenylalanine catabolic process/aromatic amino acid family metabolic process/metabolic process/oxidation reduction/response to chemical stimulus
678	Phenylalanine hydroxylase	GI:41054599	6	85	51321.8/5.6	**−2.38** *	**−1.82** *	1.31	L-phenylalanine catabolic process/aromatic amino acid family metabolic process/metabolic process/oxidation reduction/response to chemical stimulus
1344	3-hydroxyanthranilate 3,4-dioxygenase	GI:55925251	3	78	33217.5/5.54	1.21	**1.48** *	1.22	metabolic process/oxidation reduction/pyridine nucleotide biosynthetic process
***Gluconeogenesis and glycolysis (6)***									
521	Amylase, alpha 2A, pancreatic	GI:38571651	6	89	56911.7/6.43	−1.21	**−1.73** *	−1.43	carbohydrate metabolic process/metabolic process
540	Amylase, alpha 2A, pancreatic	GI:38571651	8	149	56911.7/6.43	2.14	−1.01	**−2.16** *	carbohydrate metabolic process/metabolic process
558	Amylase, alpha 2A, pancreatic	GI:38571651	8	207	56911.7/6.43	1.07	**−2.29** *	−2.46	carbohydrate metabolic process/metabolic process
593	Aldehyde dehydrogenase 8 family, member A1	GI:52218932	13	214	53319.9/6.61	**1.89** *	**2.41** *	1.27	metabolic process/oxidation reduction
638	Aldehyde dehydrogenase 8 family, member A1	GI:52218932	8	129	53319.9/6.61	**−2.09** *	**−2.48** *	−1.19	metabolic process/oxidation reduction
1213	Ldhb protein	GI:28277619	8	131	36224/6.4	−1.57	1.03	**1.61** *	carbohydrate metabolic process/cellular carbohydrate metabolic process/glycolysis/metabolic process/oxidation reduction
***Lipid metabolism (4)***									
1751	Prostaglandin D2 synthase, brain	GI:47174758	3	106	20891.2/5.24	−2.92	**−4.06** *	−1.39	lipid metabolic process/transport
1169	Sulfotransferase family 1, cytosolic sulfotransferase 3	GI:56118730	6	128	35341.4/6.55	2.25	−1.13	**−2.53** *	catecholamine metabolic process/lipid metabolic process/steroid metabolic process
1592	Apolipoprotein A-I	GI:13445027	10	199	20797/8.63	1.19	**1.49** *	1.25	lipid mobilization
1599	Apolipoprotein A-I	GI:13445027	14	329	20797/8.63	1.05	**1.46** *	**1.39** *	lipid mobilization
***TCA cycle and pyruvate metabolic (1)***									
999	PREDICTED: succinate-CoA ligase, GDP-forming, beta subunit	GI:189525094	5	112	46409.1/5.71	1.01	1.52	**1.51** *	metabolic process
***Other metabolism (4)***									
1332	Agmatine ureohydrolase	GI:117606228	4	86	39387.9/7.51	**2.18** *	1.73	−1.26	polyamine biosynthetic process
1042	Alcohol dehydrogenase 8a	GI:41223380	4	92	40545.4/8.3	−1.57	**−1.57** *	−1	alcohol metabolic process/ethanol metabolic process/metabolic process/oxidation reduction/response to chemical stimulus
578	Aldh9a1a protein	GI:44890712	4	118	55267.9/6.18	**−2.53** **	**−2.33** *	1.09	metabolic process/oxidation reduction
1513	Hypoxanthine phosphoribosyltransferase 1	GI:47085697	4	121	24682.7/6.21	**1.81** *	**1.83** *	1.01	nucleoside metabolic process/purine ribonucleoside salvage
**Electron transport (1)**									
666	ATP synthase H+ transporting mitochondrial F1 complex beta	GI:198285477	14	317	52910.6/4.87	**1.8** *	**2.3** *	1.27	ATP biosynthetic process/ATP metabolic process/ATP synthesis coupled proton transport/ion transport/proton transport/transport
**Signal transduction (3)**									
1313	Annexin A4	GI:213688814	10	213	35560.3/5.98	2.23	**2.28** *	1.02	Function: calcium ion binding, calcium-dependent phospholipid binding
1190	PREDICTED: similar to regucalcin		4	134	32816.3/5.39	2.17	**2.85** *	1.31	
115	Valosin containing protein	GI: 41393119	23	236	56911.7/5.14	−1.83	**−2.33** *	−1.27	Function: ATP binding/binding/hydrolase activity/lipid binding/nucleoside-triphosphatase activity/nucleotide binding/Process: cell cycle/transport
**Stress response (9, including oxidative stress response)**									
278	HSC70 protein	GI:1865782	9	151	71131.3/5.18	−1.26	**−1.8** *	−1.43	response to stress
256	Constitutive heat shock protein HSC70-2	GI:33598990	7	120	70550.9/5.14	1.14	**−2.31** *	**−2.64** **	response to stress
1685	Glutathione peroxidase	GI:115521902	3	96	16334.5/5.92	1.6	**1.72** *	1.07	oxidation reduction/response to oxidative stress
1589	Peroxiredoxin 6	GI:41387146	7	86	24993/6.13	1.17	**1.41** *	1.2	cell redox homeostasis
535	Sb:cb825 protein	GI:27881963	14	171	54713.7/6.32	1.29	−1.3	**−1.67** *	cell redox homeostasis
87	Heat shock protein 90 kDa beta, member 1	GI:38016165	14	224	91224.9/4.77	−1.59	**−2.11** **	−1.33	protein folding/response to stress
238	Heat shock protein 5	GI:39645428	21	410	72946.2/5.04	1.13	−1.35	**−1.52** *	response to stress
1045	Mitochondrial ATP synthase beta subunit	GI:147905995	8	115	55779.4/5.19	−1.42	1.27	**1.8** *	ATP biosynthetic process/ATP metabolic process/ATP synthesis coupled proton transport/ion transport/proton transport/transport
669	RecName: Full = ATP synthase subunit beta, mitochondrial, Flags: Precursor	GI:47605558	15	329	55212.9/5.05	1.33	**1.88** *	**1.41** *	ATP biosynthetic process/ATP metabolic process/ATP synthesis coupled proton transport/ion transport/proton transport/transport
**Cytoskeleton (9)**									
488	Tubulin beta-2C chain	GI:223647034	11	256	49747.9/4.76	**−2.53** *	**−2.26** *	1.12	microtubule-based movement/microtubule-based process/protein polymerization
607	Alpha tubulin	GI:10242166	6	114	45599.5/5.65	−1.66	**−1.67** *	1.01	KEGG Ontology: cytoskeleton/cytoskeleton proteins
443	PREDICTED: similar to Tubulin beta-6 chain (Beta-tubulin class-VI)	GI:125819301	11	168	52544.5/4.9	**1.46** *	**1.42** *	−1.03	KEGG pathway: gap junction
843	Beta actin	GI:27805142	10	263	41707.6/5.29	−1.13	2.17	**2.46** **	KEGG Ontology: cytoskeleton/cytoskeleton proteins
820	Keratin 8	GI:41056085	11	259	57723.4/5.15	1.64	2.22	1.35	cytoskeleton/cytoskeleton proteins
332	Plastin 3	GI:50539712	3	96	70105.2/5.95	−1.13	1.93	**2.18** *	Function: actin binding/calcium ion binding
750	Keratin-like protein	GI:226510657	17	319	42477.4/4.85	2.85	**3.41** *	1.19	
714	Spna2 protein	GI:62132941	9	214	55447.4/5.04	2.05	**2.97** *	1.45	clustering of voltage-gated sodium channels
663	Type I cytokeratin, enveloping layer	GI:41388915	6	88	46524.6/5.13	1.95	**2.14** *	1.09	cell migration involved in gastrulation
**Protein translation and maturation (3)**									
213	Protein disulfide isomerase A4	GI:41054259	8	100		−1.31	**−2.52** *	**−1.93** *	cell redox homeostasis
832	40S ribosomal protein SA	GI:41054259	3	127	21590.9/8.2	−1.31	**−1.85** *	−1.41	
1445	Ubiquitin carboxyl-terminal esterase L3 (ubiquitin thiolesterase)	GI:66773134	5	76	25910.1/4.88	**1.68** *	−1.2	**−2.01** *	
**Other functions (7)**									
550	Calreticulin precursor	GI:224613524	6	97	44632.2/4.42	**2.29** *	**1.82** *	−1.26	
393	Procollagen-proline, 2-oxoglutarate 4-dioxygenase (proline 4-hydroxylase), beta polypeptide	GI:193788703	6	155	56598.2/4.55	1.24	**1.67** *	1.35	genetic information processing/folding, sorting and degradation/chaperones and folding catalysts [KEGG]
315	Transferrin variant F	GI:189473163	11	199	73040.6/5.91	−2.43	**−11.6** *	−4.78	
327	Transferrin variant D	GI:189473159	11	205	73137.5/5.77	−4.13	**−14.54** *	**−3.52** *	
1399	Zgc:56585 protein	GI:42744582	3	124	29058/5.2	−1.15	**2.27** *	**2.62** **	
1396	Zgc:56585 protein	GI:42744582	4	80	29058/5.2	**−1.48** *	1.39	**2.05** **	
616	Unnamed protein product	GI:47218629	13	328	55109/5.09	−1.26	1.03	**1.3** *	

a Unique spot number of the position where the spot is displayed in the master gel.

b Accession number according to the NCBI rat database.

c Number matched peptides.

d Protein scores greater than 64 were successfully identified.

e Fold-change between the T2, T1 and C groups.

*p<0.05 was considered statistically significant and

**p<0.01 was extremely significant.

A positive value signifies up-regulation and a negative value signifies down-regulation. C: laboratory (Control), T1: Xukou Bay, T2: Meiliang Bay.

f The identified proteins were grouped according to their functions based on the Gene Ontology and KEGG databases.

### Biological function of identified protein

**Figure 5:**
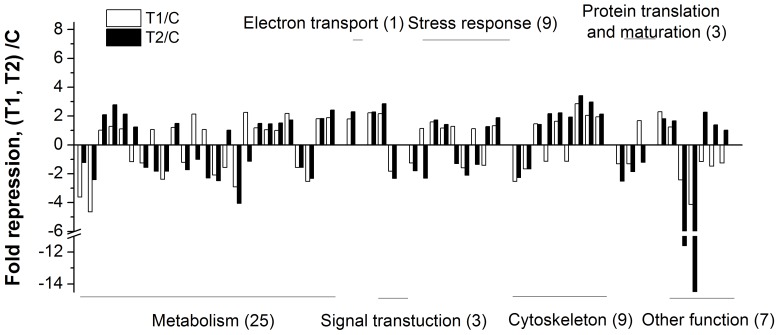
Expression level and classification of differentially expressed proteins in the liver of *C. carpio* collected from different *in situ* experiment sites. T1: Xukou Bay; T2: Meiliang Bay.

**Figure 6:**
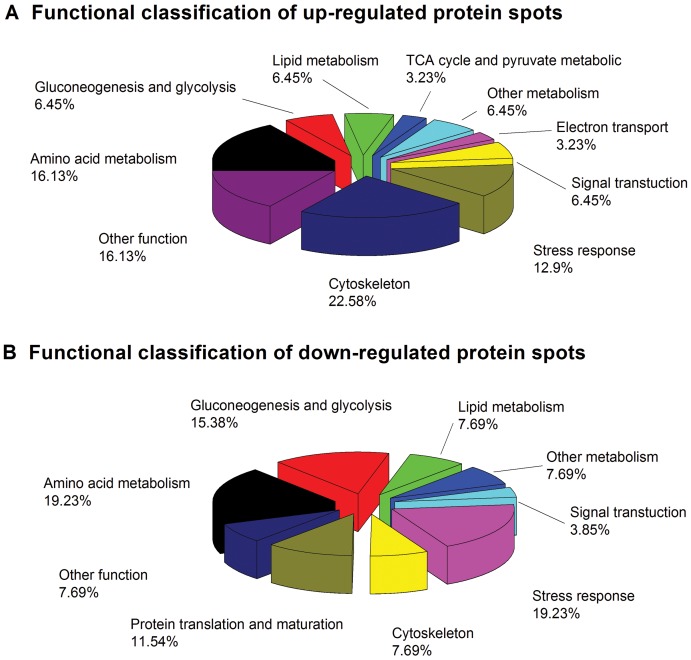
Functional classification of up-regulated (A)/down-regulated (B) proteins from the liver of *C. carpio* in the T2 group.

The biological functions of the proteins were analyzed based on the GenBank, Gene Ontology and KEGG databases to reveal the potential toxic mechanism underlying cyanobacterial blooms (Table 2). The classification and up- and down regulation of these proteins are shown in Figure 5. Among the 57 identified proteins, 25 proteins (43.9%) were involved in metabolic processes. Among these, 10 proteins were involved in amino acid metabolism, 6 proteins were involved in glucose metabolism and 4 proteins were associated with lipid metabolism. In addition, 9 proteins were associated with the cytoskeleton reorganization and stress. Among 57 proteins identified, 23 proteins were up-regulated and 19 proteins were down-regulated among the carp cultured at different experiment sites. Changes greater than 1.5-fold were observed in 45 of the 57 proteins identified. Compared with the control and T1 groups, a significant difference in the protein expression was observed in the T2 group. The functional classification of significantly up/down-regulated proteins from the liver of *C. carpio* in the T2 group is shown in Figure 6. A total of 31 up-regulated proteins were involved in metabolic processes, cytoskeleton reorganization, and stress defense, while 26 down-regulated proteins were involved in metabolic processes, stress defense, protein translation and protein modifications.

### Quantitative PCR and Western blot

Using real-time quantitative PCR, the mRNA levels of the oxidative stress-related enzyme glutathione peroxidase (GP), the mitochondrial stress protein mitochondrial ATP synthase beta subunit (MASB) and the signal transduction protein valosin-containing protein (VCP) were detected. GADPH was used as a control. As shown in Figure S2, significant differences were observed in the T2 group compared with other groups (*p*<0.05). The levels of VCP and MASB mRNA were well correlated with the protein expression levels. However, the level of GP mRNA was not correlated with the GP expression level, suggesting that protein expression might not be associated with the transcript level and could therefore be affected through post-transcriptional modifications [21].

As shown in Figure S3, the Western blot data were consistent with the data obtained from the 2D-DIGE. Antibodies against beta-tubulin and HSP70 were used for Western blotting, and the results indicate that 2D-DIGE was reliable for analyzing differential protein expression.

## Discussion

With the development of industry and agriculture, the advanced eutrophication in Lake Taihu, particularly in Meiling Bay, poses a serious threat to the sustainability of freshwater ecosystems. Cyanobacterial blooms indicate advanced eutrophication, and the presence of these organisms is increasing worldwide. Microcystins are the most common cyanobacterial toxins detected in water and are most often responsible for poisoning animals and humans [2]. Although extensive studies have focused on the mechanisms underlying the toxicity of MCs, the co-toxic effects of the microcystins with other pollutants remain unknown. Further examination of the effects of a variety of pollutants on the biological ecotoxicology of organisms is greatly needed [22].

*Microcystis aeruginosa* are the dominant species in Meiling Bay. The highest algal density (2.35×10^5^ cells/mL) was detected at site S3. Cyanobacterial bloom outbreaks in Lake Taihu were significantly associated with water temperature, TP and MC-LR/RR concentration (*p*<0.05, *p*<0.01). The water temperature and TP directly affected cyanobacterial blooms. Of the tissues tested, MC accumulation was highest in the liver of carp cultured at site S1 (256.2 ng g^−1^), which was much higher than that in the carp (59.9 ng g^−1^) cultured in water containing 0.334/0.184 µg L^−1^ of MC-LR/RR [16]. Notably, the dissolved MC-LR/RR (0.334/0.184 µg L^−1^) was much lower in the *in situ* experiments than that observed under laboratory experimental conditions (10 µg L^−1^ of MC-LR). Although the numerous algae observed in the intestine of cage-cultured carp in Meiliang Bay indicates that food intake might be important for the accumulation of MCs, wild fish might not uptake lots of cyanobacterial cells due to food selectivity or food aversion. Many studies have reported the accumulation and distribution of MCs in different tissues/organs in various species of fish [23]–[28]. Xie et al. [27] reported that the MC levels detected in the liver, intestine and muscle of carp captured from Chaohu were higher than the TDI value of WTO (0.04 g kg^−1^ d^−1^). In the present study, the level of MCs was lower than the TDI value. The MC levels were significantly associated with a variety of factors, such as temperature, pH, cyanobacterial bloom, electrical conductivity, and heavy metal ions. This result indicates that many factors affect the accumulation of MCs in the liver. Moreover, the presence of metal ions might promote the absorption of MCs in fish.

Previous studies have indicated that the ROS levels are associated with exposure time and MC-LR levels [16], [29]. We observed that the ROS levels were significantly higher in the carp cultured in Meiling Bay, than in those cultured in Xukou Bay and the control group. Moreover, the ROS levels were closely associated with the MC levels in the liver, suggesting that the ROS levels, associated with water eutrophication, could be used as a biomarker.

The effects of cyanobacterial blooms on the proteomic changes in liver toxicology have not received much attention until recently [30]–[34]. In the present study we also considered the effects of environmental factors, nutrient concentrations, the levels of organic and heavy metals on liver toxicology. Table 2 lists the identified proteins involved in multiple biological processes, such as metabolic processes, stress defense, cytoskeletal protein, protein translation and protein processing. Most of the proteins identified are involved in metabolism processes, including amino acid metabolism, gluconeogenesis and glycolysis, and lipid metabolism, which is an important function of the liver. The significant up-regulation of protein in the T2 group (Meiliang Bay) included histidine ammonia-lyase (HAL), LOC556744, methionine adenosyl transferase (MAT), and 3-hydroxyanthranilate 3,4-dioxygenase (AAO), which are involved in amino acid metabolism. The down regulation of 1,2-dioxygenase (HGD) and phenylalanine hydroxylase (PaH) was observed in the T2 group. Only fumarylacetoacetase and PaH were up-regulated in the T1 group (Xiukou Bay) compared with the T2 group. These data indicate that exposure to cyanobacterial blooms *in situ* could interfere with the metabolism of amino acids in the liver.

The down regulation of proteins involved in gluconeogenesis and glycolysis was observed in both the T1 and T2 groups. Alpha amylase 2A enzyme is a key enzyme involved in the initial stages of the hydrolysis of oligosaccharides and polysaccharides [35]. The expression of alpha-amylase 2A and pyruvate kinase could be significantly inhibited through MC-LR [36]. Mezhoud et al. [37] reported that the MC-LR could decrease the glycogen content in the liver and increase the biological consumption of energy. Consistent with the results of the present study, the significant inhibition of alpha-amylase 2A was observed in the T2 group.

The up-regulation of succinyl-CoA ligase (Suclg) was observed in the T2 group, which could promote the tricarboxylic acid (TCA) cycle through the decomposition of succinyl-CoA and guanosine diphosphate (GDP) into succinic acid and ATP in the carp liver. The increase in amino acid metabolism could promote pyruvate metabolism and the TCA cycle, as many of the metabolites from amino acids can directly enter into the TCA cycle, consistent with the toxicological effects of MC-LR on zebrafish [36]. In addition, a protein involved in the electron transport biological pathways was observed in the T2 group, indicating that energy metabolism was increased after exposure to cyanobacterial blooms.

Excess ROS were produced in the liver after exposure to cyanobacterial blooms, which could further induce lipid peroxidation. The changes of proteins involved in lipid metabolism, including prostaglandin D2 (PTGDS), cytosolic sulfotransferase 3 (SULT3), and apolipoprotein AI (ApoAI) were observed. Notably, the effects of MC-LR on PTGDS and SULT3 were first reported in the present study. The up-regulation of ApoAI in the liver is consistent with previous studies of Japanese medaka as reported by Malécot et al. [38]. ApoAI plays an important role in lipoprotein metabolism by affecting the activity of an enzyme associated with lipoprotein metabolism, binding to a lipoprotein ligand, and inhibiting metabolic processes of lipoprotein receptors.

Changes in cytoskeleton-associated proteins, such as tubulin beta-2D, alpha tubulin, keratin-like protein, Spna2 protein, and Type I cytokeratin, were observed in the T2 group compared with the other groups. Tubulin and actin are essential for maintaining cell structure integrity and function. Studies have confirmed that MC-LR cytotoxicity resulted from the regulation of cytoskeletal protein expression and caused the destruction of the cytoskeleton [36], [38], [39]. In a previous study, we also showed that MC-LR could induce the reorganization of liver cytoskeletal proteins in the carp. MC accumulation in the liver inhibited the activity of protein phosphatases (PP1 and 2A) and induced cytoskeletal reorganization. In addition, MCs bind to tubulin and destroy the balance of polymerization/depolymerization [39]. Notably, the damage to cytoskeletal proteins could inhibit the transport of transferrin and induce the secretion of apolipoprotein AI (ApoAI) [36], [40], consistent with the down regulation of transferrin and the up-regulation of ApoAI in the T2 group.

As shown in Figure 5 and Table 2, changes in defense-related proteins, including heat shock proteins (HSPs) and oxidative stress-related proteins, were observed in the T2 group but not the T1 group. This result indicates that these proteins are associated with cyanobacterial blooms. The up-regulation of defense-related proteins included glutathione peroxidase (GP), peroxiredoxin 6 (Prdx 6) and mitochondrial ATP synthase beta subunit (MASB), whereas the down regulation of defense-related proteins included HSC70 protein, HSC70-2 and HSP90 beta class B member1. Peroxiredoxin (Prdx) is a novel family peroxidase that exists in six different isoforms. Some of these isoforms provide defense against oxidative damage, whereas other isoforms participate in signaling pathways through the regulation of the H_2_O_2_ concentration [41]. Chen et al. [42] reported the significant up regulation of Prdx 2 and down regulation of Prdx1 and Prdx6 in MC-LR treated mice. Prdxs has anti-apoptotic functions [43] and inhibits tumor growth through the inhibition of H_2_O_2_ [44]. Therefore, the up regulation of PRDX 6 in the T2 group might promote the observed antioxidant and anti-apoptotic effects. HSPs are induced under stress conditions and protect cells from damage. The down regulation of HSPs was observed in the present study, suggesting that MCs might inhibit HSP expression and function. One of the mechanisms of MC toxicity involves binding to MASB and inducing oxidative stress through the interference of mitochondrial membrane potential and permeability transition [45], suggesting that the changes in MASB are induced through MCs. In addition, Malécot et al. [38] reported that changes in ALDH expression are associated with oxidative stress. The data obtained in the present study strongly suggest that oxidative stress plays an important role in MC toxicity in the carp after exposure to cyanobacterial blooms.

Changes in the proteins involved in signal transduction pathways, including annexin A4 and VCP protein, were also observed in the T2 group. Annexin A4 exhibits anti-apoptotic activity and plays an important role in signal transduction and the negative regulation of cohesion through binding to calcium or calcium-dependent phospholipids. In addition, studies have shown that Annexin A4 plays a critical role in heat and oxidative stress and might also be involved in the ROS pathway [36], [46]. VCP is a widespread membrane-binding glycoprotein that plays an important role in the degradation of endoplasmic reticulum-associated proteins and cell cycle regulation.

Based on the proteomics data, the protein changes induced through cyanobacterial bloom formation might be one of the toxic mechanisms of MCs. Malécot et al. [38] reported that the mechanism underlying MC-LR toxicity involved the transportation of this toxin into liver cells through OATP (an organic anion transporting polypeptide), which inhibited the expression of PP1, PP2A and ALDH, the reorganization of cytoskeletal proteins, oxidative stress, etc. This inhibition would further cause DNA damage, eventually leading to the apoptosis of the cell. Similar effects were also observed with cyanobacterial bloom exposure in the present study. In addition, changes in the expression of proteins, such as ALDH, MASB, 40S ribosomal protein SA, etc., were observed not only in the cytoplasm but also in the mitochondria and endoplasmic reticulum, suggesting that oxidative, mitochondrial and endoplasmic reticulum stress also play an important role in MC toxicity.

## Conclusion

In the present study, we present the first analysis of the protein profiles in the liver of *C. carpio* exposed to cyanobacterial blooms using the 2D-DIGE. MCs were absorbed through the gills and intestine and subsequently transported to liver cells, which induced the up regulation of liver ROS levels and inhibited the expression of PP1, PP2A and ALDH. MC accumulation also induced oxidative stress and the reorganization of the cytoskeleton, thereby affecting a series of related proteins and interfering with carp liver metabolic pathways. The accumulation of MCs enhanced amino acid metabolism and the TCA cycle and inhibited glucose metabolism. These effects might be associated with both microcystins and ammonia nitrogen stress. These data provided evidence of the molecular mechanisms involved in liver damage in fish exposed to cyanobacterial blooms.

## Supporting Information

Table S1 Experimental design for the 2-D-DIGE analysis.
(DOC)

Figure S1 2D DIGE gel image of *C. carpio* (24 cm IPG strip, pH 4–7, gel 2). (A) Cy2 labeled IS sample; (B) Cy3-labeled X group; (C) Cy5-labeled M group; (D) 3-channel coincidence image.
(TIF)

Figure S2 Quantitative PCR analysis of renal mRNA expression levels of GP, MASb and VCP from carp cultured in the laboratory (Lab), Xukou Bay (T1) and Meiliang Bay (T2), respectively. The values indicate the means ± SD (n = 4). *p<0.05.
(TIF)

Figure S3 Effects of field treatment on β-tubulin (A) and HSP70 (B) expression in the liver of *C. carpio*, including a representative autoradiograph of the WB. Equal protein loading was confirmed using the anti-β-actin antibody. The data were normalized to the β-actin signal. The values indicate the means ± SD (n = 4). *p<0.05.
(TIF)

Text S1 Protocols for the quantitative PCR and Western blot analysis.
(DOC)
