# Supplementary material for: Proteomic Analysis of Hepatic Tissue of Cyprinus carpio L. Exposed to Cyanobacterial Blooms in Lake Taihu, China
Source: PLoS One. 2014 Feb 18;9(2):e88211. doi: 10.1371/journal.pone.0088211 (PMC3928196; doi:10.1371/journal.pone.0088211)
Supplement: Table S1 — Experimental design for the 2-D-DIGE analysis. (DOC) [file pone.0088211.s001.doc]

**Table S1.** Experimental design for 2-D-DIGE analysis.

| Gel no. | CyDye^TM^ DIGE Fluor minimal dyes | | |
| --- | --- | --- | --- |
|  | Cy2 | Cy3 | Cy5 |
| 1 | IS ^a^ （50 μg ） | C1 ^b^（50 μg ） | X1（50 μg ） |
| 2 | IS | X2 | M1 |
| 3 | IS | M2 | C2 |
| 4 | IS | C3 | X3 |
| 5 | IS | M3 | C1 |

^a^ Internal Standard (IS) pooled from equal amount of nine experimental samples; ^b^ C1-C3 were protein samples from control fish in laboratory, X1-X3 were protein samples of cage-cultured fish in Xukou Bay, M1-M3 were protein samples of cage-cultured fish in Meiliang Bay. Each experimental sample was pooled from three randomly selected liver samples in the same group. Gels were loaded with paired samples according to the Table S1.
