# Supplementary figures and images for: Proteomic Analysis of Hepatic Tissue of Cyprinus carpio L. Exposed to Cyanobacterial Blooms in Lake Taihu, China
Source: PLoS One. 2014 Feb 18;9(2):e88211. doi: 10.1371/journal.pone.0088211 (PMC3928196; doi:10.1371/journal.pone.0088211)

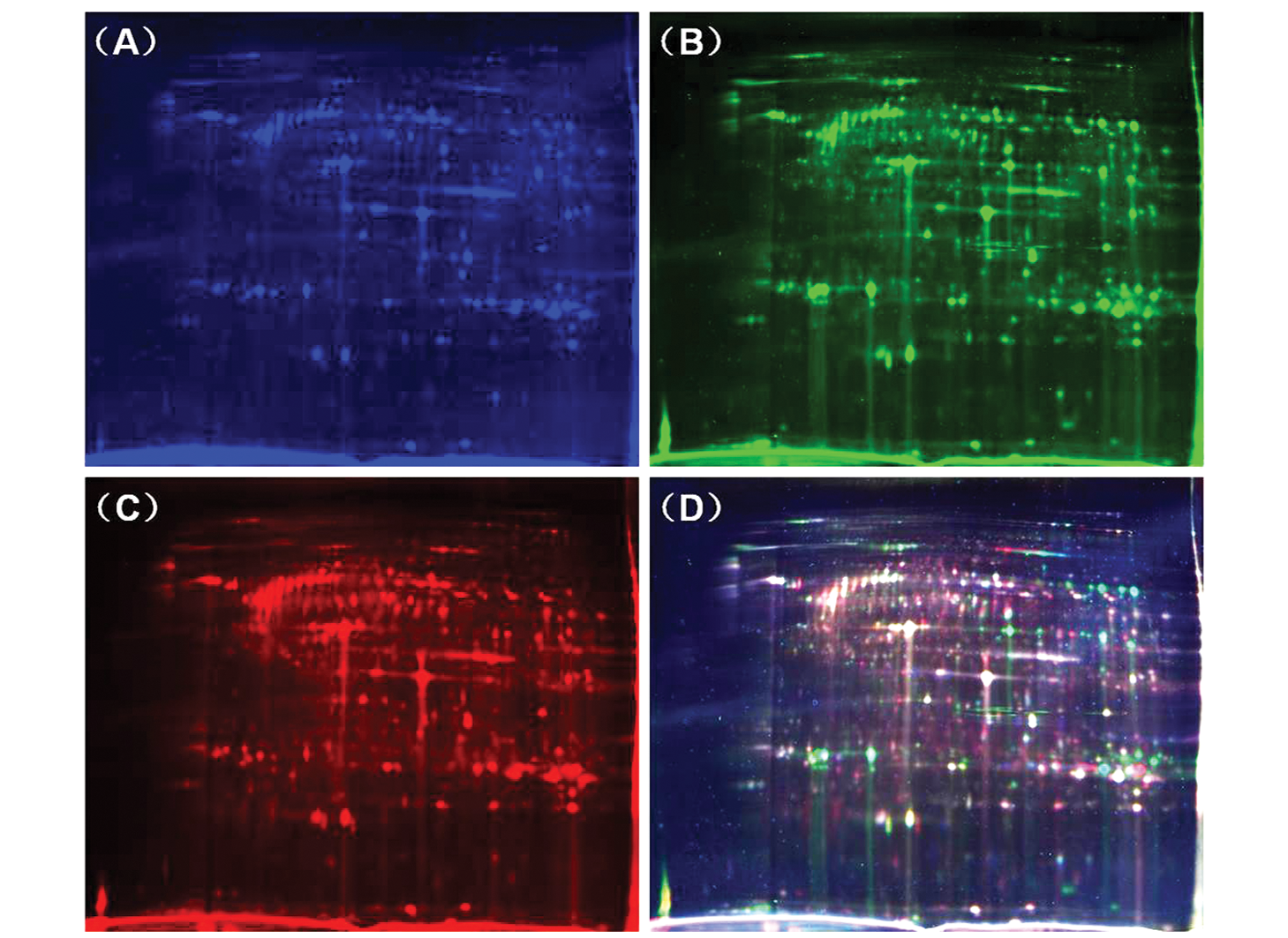

Supplement: Figure S1 — 2D DIGE gel image of C. carpio (24 cm IPG strip, pH 4–7, gel 2). (A) Cy2 labeled IS sample; (B) Cy3-labeled X group; (C) Cy5-labeled M group; (D) 3-channel coincidence image. (TIF) [file pone.0088211.s002.tif]

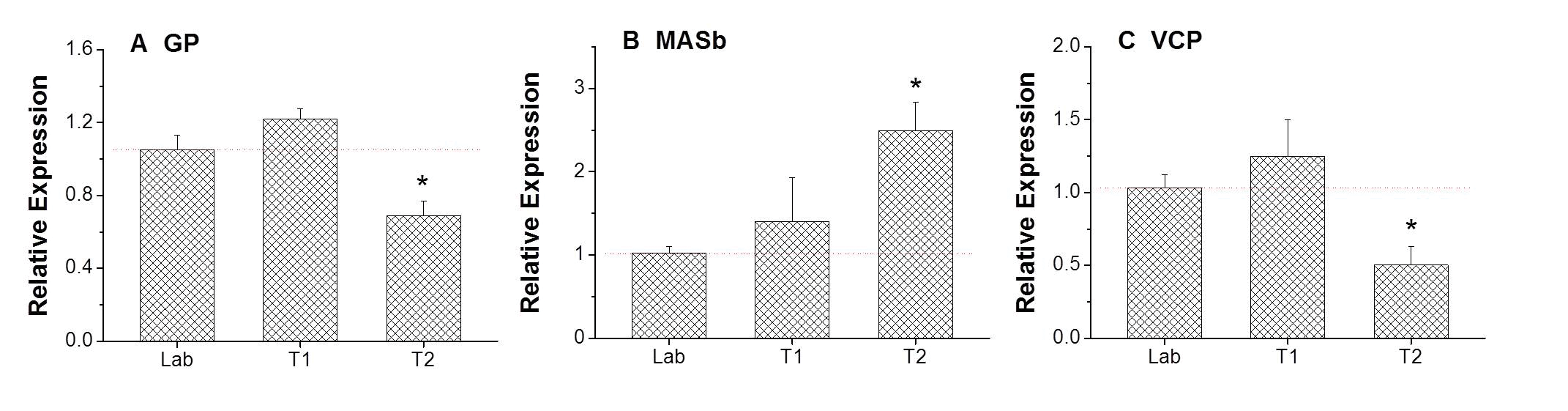

Supplement: Figure S2 — Quantitative PCR analysis of renal mRNA expression levels of GP, MASb and VCP from carp cultured in the laboratory (Lab), Xukou Bay (T1) and Meiliang Bay (T2), respectively. The values indicate the means ± SD (n = 4). *p<0.05. (TIF) [file pone.0088211.s003.tif]

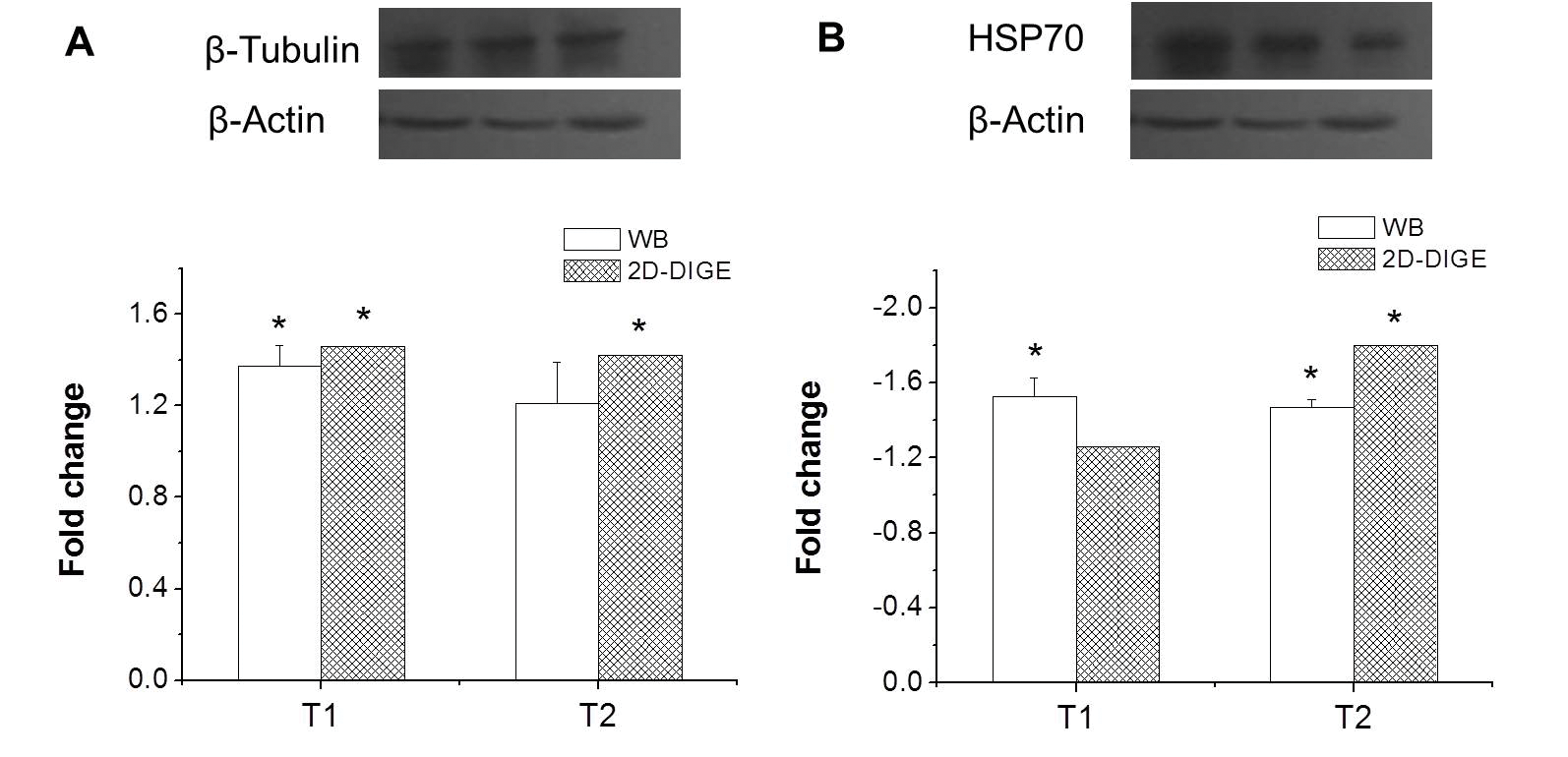

Supplement: Figure S3 — Effects of field treatment on β-tubulin (A) and HSP70 (B) expression in the liver of C. carpio, including a representative autoradiograph of the WB. Equal protein loading was confirmed using the anti-β-actin antibody. The data were normalized to the β-actin signal. The values indicate the means ± SD (n = 4). *p<0.05. (TIF) [file pone.0088211.s004.tif]
