# Supplementary material for: Proteomic Analysis of Hepatic Tissue of Cyprinus carpio L. Exposed to Cyanobacterial Blooms in Lake Taihu, China
Source: PLoS One. 2014 Feb 18;9(2):e88211. doi: 10.1371/journal.pone.0088211 (PMC3928196; doi:10.1371/journal.pone.0088211)
Supplement: Text S1 — Protocols for the quantitative PCR and Western blot analysis. (DOC) [file pone.0088211.s005.doc]

**Text S1**

**Supplementary Methods**

**Real-time quantitative PCR.** Total RNA was isolated from liver samples using the TRIzol kit (Invitrogen) according to the manufacturer’s instructions. The isolated total RNA was treated with DNAase to eliminate residual DNA prior to reverse transcription of total RNA to complementary DNA (cDNA). The RNA concentration was measured by absorbance at 260 nm. The RNA purity was estimated by the 260/280 nm absorbance ratio. One microgram of total RNA was characterized by denatured agarose gel electrophoresis. Primers for the evaluation of expression of genes VCP (forward primer: 5’-GTGGACATCGGCATTCCAGA -3’; reverse primer: 5’-AGCCACCTGCTCCAGATCAAC-3’), MASb (forward primer: 5’- AATGTCATCGGTGAGCCCATT-3’; reverse primer: 5’-AATCTCCTGCTCGACGCTCAT -3’), GP (forward primer: 5’-AACGCCCACCCTCTGTTTG-3’; reverse primer: 5’-TCCTGT TCACCGGACTCCAG-3’), and 18 S rRNA (forward primer: 5’-TTGTTGGTGTTGTTGCT GGT-3’; reverse primer: 5’-GGATGCTCAACAGGGGTTCAT-3’) were designed using Primer Express 2.0 (Applied Biosystems). The cDNA was synthesized with 0.5 μg of total RNA using the PrimerScript RT reagent Kit (TaKaRa) on a GeneAmp® PCR System 9700 (Applied Biosystems) according to the manufacturer’s protocol.

Quantification of gene expression was carried out on a 7500 Fast Real-Time PCR System (Applied Biosystems). The reaction mixture was composed of 10 μl of SYBR Green Realtime PCR Master Mix (TaKaRa), forward and reverse primers (10 μM, 0.4 μl each), ROX Refrence Dye II (50×, 0.4 μl), 7.8 μl of nuclease-free water, and the cDNA sample (1 μl). The PCR protocol was 95 ºC for 15 seconds followed by 40 cycles of 95 ºC for 5 seconds and 62 ºC for 34 seconds. After PCR, a melting curve analysis was performed to obtain the specificity of the PCR product, which was displayed as a single peak. Every sample was analyzed in triplicate. Differences in expression levels were calculated using the 2-∆∆Ct method (Pfaffl, 2001). Amplification of 18S rRNA (Applied Biosystems) was used as an internal control for VCP, MASb, and GP expression, which was also used in other studies (Žegura et al., 2008). Statistical significance was determined using a one-way ANOVA followed by the Duncan multiple range test (SPSS, Inc., Chicago, IL). Data are presented as means with standard errors (mean ± SE). A *p*-value < 0.05 was considered as statistical significance.

**Western blot.** The independent protein samples extracted for DIGE experiment (four per group) were used for western blot analysis to validate the results of 2D-DIGE. The proteins were denatured at 95°C for 5 min, and 25 µg of protein was separated by SDS-PAGE (5% stacking gel and 10% resolving gel) and then transferred onto the polyvinylidene ﬂuoride (PVDF) membrane. After blocking by non-fat milk (8%), the PVDF membrane was incubated with primary antibodies including rat β-Tubulin monoclonal antibodies (ZSGB-BIO), rabbit monoclonal antibodies to fish HSP70 (Stressgen) and rat β-Actin monoclonal antibodies (LifeTein) at 4°C overnight, respectively. Antibody concentrations were determined on the basis of the manufacturer’s recommendations. After incubated with primary antibodies, membranes were washed and incubated with an appropriate horseradish peroxidase (HRP)-conjugated secondary antibody (dilution 1:10000 in 1% BSA/TBS-T). Then the protein bands were visualized by enhanced chemiluminescence (superECL, Tigen, Beijing, China). The densitometry analyses were conducted using Image J software. Data were normalized to protein expression levels of β-Actin. Statistical significance was determined using a one-way ANOVA followed by the Duncan multiple range test (SPSS, Inc., Chicago, IL). Data are presented as mean ± SD and *p* < 0.05 was considered significant.
